# Caveolin-1 deficiency improved glucose metabolism via modulation of β-cell autophagy in high-fat diet-fed mice

**DOI:** 10.1016/j.jbc.2026.111480

**Published:** 2026-04-22

**Authors:** Wen Zeng, Nan Cai, Jia Liu, Kunying Liu, Shuo Lin, Xubin Yang, Longyi Zeng

**Affiliations:** 1 Department of Endocrinology & Metabolism, The Third Affiliated Hospital of Sun Yat-sen University, Guangzhou, Guangdong, China; 2 Guangdong Provincial Key Laboratory of Diabetology, The Third Affiliated Hospital of Sun Yat-sen University, Guangzhou, Guangdong, China; 3 Guangzhou Municipal Key Laboratory of Mechanistic and Translational Obesity Research, The Third Affiliated Hospital of Sun Yat-sen University, Guangzhou, Guangdong, China; 4 Medical Center for Comprehensive Weight Control, The Third Affiliated Hospital of Sun Yat-sen University, Guangzhou, Guangdong, China

**Keywords:** autophagy, caveolin-1, palmitate, pancreatic β cells, insulin resistance, glucose tolerance, apoptosis

## Abstract

Lipotoxicity caused β-cell mass decrease and impaired β-cell function in type 2 diabetes mellitus. We previously reported that caveolin-1 (Cav-1) deficiency protected pancreatic β cells against palmitate (PA)-induced apoptosis and dysfunction in both NIT-1 cells and isolated islets. In this study, we firstly established inducible β-cell-specific Cav-1 KO mice model. Next, we investigated whether Cav-1 depletion *in vitro* or *in vivo* affected β-cell function and survival through the regulation of autophagy under lipotoxicity. Our results showed that Cav-1 depletion exhibited increased islets size, improved insulin resistance and glucose tolerance in lipid stressing conditions. In addition, KO of Cav-1 also increased β-cell viability through suppression of gene expression of pro-apoptotic molecules (BIM, BID, SMAC, Apaf, Caspase9, Caspase3, AIF, and EndoG). Mechanism studies revealed that Cav-1 depletion protected β cells from PA-induced apoptosis through p38 MAPK signaling pathway. Meanwhile, we also found that Cav-1 depletion enhanced autophagy through elevated expression of Beclin-1 and Lc3b, and increased degradation of p62 protein. Further investigation indicated that mechanistic target of rapamycin (mTOR) signaling pathway was participated in the regulation of PA-induced autophagy. Taking together, our data suggested that Cav-1 depletion ameliorated PA-induced glucose metabolism abnormality, increased cell viability, and improved β-cell apoptosis through the enhancement of autophagy. Our findings would provide valuable clues for developing novel treatments based on Cav-1 inhibition against diabetes under lipotoxicity.

The prevalence of diabetes was estimated to be 589 million in 20 to 79 years adults in 2024 and would continue to increase to 853 million in 2050 worldwide (1). Overweight/obesity is a major factor leading to the development of type 2 diabetes mellitus. The pathogenesis of this process might involve in β-cell dysfunction and apoptosis under free fatty acids (FFAs)-induced lipotoxicity (2). However, how apoptosis and dysfunction of the β cells is triggered is largely unknown, presenting challenges for effective diabetes prevention and treatment.

Autophagy is a lysosomal degradative process of damaged organelles, lipids, and dysfunctional proteins, which is crucial for the maintenance of cellular homeostasis (3, 4, 5). Recently, autophagy is found to facilitate proper β-cell function and survival under cell stressed environment including FFAs overloading (6, 7, 8). FFAs treatment often exhibited dysregulated autophagy activity with impaired β-cell glucose-stimulated insulin secretion (3, 9, 10), while measures facilitating autophagy exerted protective effects against β-cell dysfunction (11, 12). Thus, proper enhancement of β-cell autophagy might be beneficial to the development of effective interventions for diabetes treatment.

Caveolin-1 (Cav-1) is a principal structural component of plasma membrane invaginations called caveolae. Previous studies demonstrated that Cav-1 is critical to insulin receptor-mediated signaling, insulin secretion, and potentially the development of diabetes (13, 14, 15, 16). Particularly, Cav-1 KO mice showed diabetic symptoms of postprandial hyperinsulinemia, insulin resistance (17, 18), β-cell death decrease (19, 20, 21), and resistance to hypertriglyceridemia (22, 23). In addition, our *in vitro* studies verified that Cav-1 silencing promoted β-cell proliferation, induced insulin secretion, and improved NIT-1 β-cell apoptosis under lipotoxicity (24).

Recent studies demonstrated that Cav-1 might participate in regulating autophagy in various cell types (25, 26, 27, 28, 29). Besides, Cav-1 was found to facilitate low-density lipoprotein transcytosis and regulate autophagy in cell stress responses (30, 31, 32). Our previous research also found that Cav-1 is important in regulating lipotoxicity-induced β-cell intracellular lipid accumulation and inflammation (33). However, whether Cav-1 is associated with autophagy regulation in β cells under lipotoxicity is unknown. In this study, we firstly generated Cav-1 depleted NIT-1 cell model and an inducible β-cell-specific Cav-1 KO mice model. Then, we found that Cav-1 deficiency improved palmitate (PA)-induced glucose metabolism abnormality, increased cell viability, and improved β-cell apoptosis through the enhancement of autophagy.

## Results

### Cav-1 depletion increased islets size without causing morphological abnormalities

**Figure 1:**
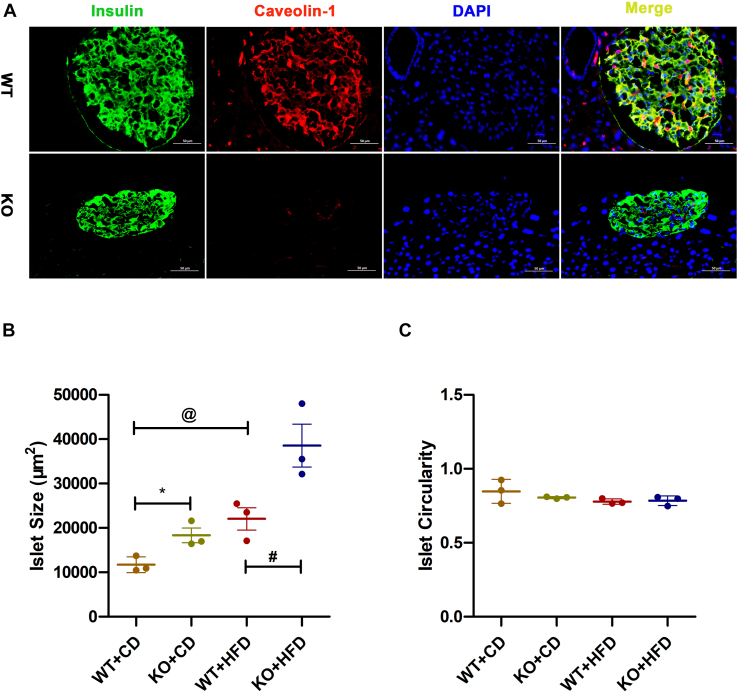
**Study of islet morphology.***A*, immunofluorescence staining of Caveolin-1 expression in islets from WT and β-Cav-1 KO mice (200 × magnification, the scale bar represents = 100 μm). Insulin is stained *green*, Cav-1 is stained *red*, cell nuclei are stained *blue*, and the merged staining of insulin and Cav-1 appears *yellow*. *B*, islets size and *C*, islets circularity is presented (n = 3). Data are shown as individual data points along with their respective means. ∗ = *p* < 0.05 between WT + CD group and KO + CD group; ^@^ = *p* < 0.05 between WT + CD group and WT + HFD group; ^#^ = *p* < 0.05 between WT + HFD group and KO + HFD group. CD, control diet; HFD, high-fat diet; β-Cav-1 KO, β-cell-specific Cav-1.

To study the effect of Cav-1 expression on β-cell function regulation under high-fat diet (HFD), β-cell-specific Cav-1 KO (β-Cav-1 KO) mouse model was generated as described in our previous study (33). Successful knock out of Cav-1 in mouse islets was confirmed by immunofluorescence staining (Fig. 1*A*). We then evaluated the morphological change of the islets in all groups of mice after 16 weeks on control diet (CD) or HFD. Our results showed that the islet size in the WT group increased significantly on HFD (WT + CD/11710 ± 1776 *versus* WT + HFD/22046 ± 4373 μm^2^; *p* = 0.019). Furthermore, islet size was significantly larger in KO mice compared to WT mice on both the CD (WT + CD/11710 ± 1776 *versus* KO + CD/18334 ± 2828 μm^2^; *p* = 0.026) and HFD (WT + HFD/22046 ± 4373 *versus* KO + HFD/38563 ± 8363 μm^2^; *p* = 0.039) (Fig. 1*B*). However, diet types or Cav-1 expression did not affect islets circularity, leading to no change on functional abnormalities (Fig. 1*C*). Taken together, morphological results suggested that Cav-1 depletion facilitated mice produce lager while healthy islets in reaction to higher lipid intake.

### Pre-exposure KO of β-Cav-1 prevented the increase in blood glucose levels and homeostatic model assessment of insulin resistance (HOMA-IR) induced by HFD in mice

**Figure 2:**
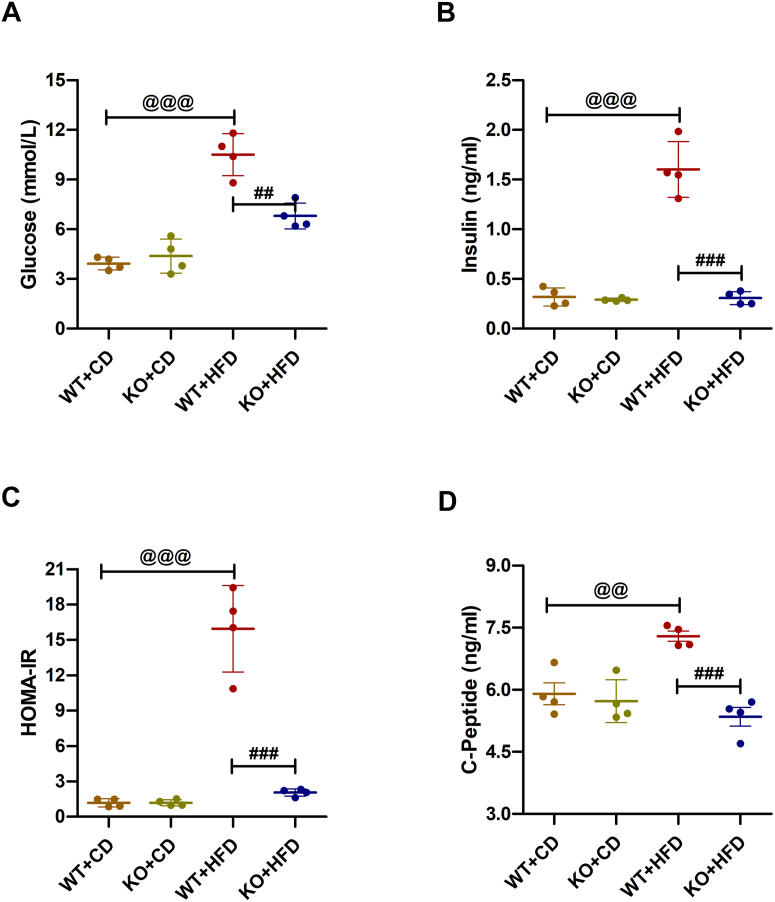
**Measurement of serum glucose, insulin, HOMA-IR, and C-peptide in pre-exposure Cav-1 knockout study.** Serum levels of basal glucose (*A*), basal insulin (*B*), HOMA-IR (*C*), and basal C-peptide (*D*) in wild-type (WT) and pre-exposure knockout of Cav-1 mice fed a control diet (CD) or high-fat diet (HFD) were measured according to each kit’s instructions. The data are representative of four independent experiments and represent the mean (n = 4) ± SD. ^@@^ = *p* < 0.01 and ^@@@^ = *p* < 0.001 between WT + CD group and WT + HFD group; ^##^ = *p* < 0.01 and ^###^ = *p* < 0.001 between WT + HFD group and KO + HFD group. HOMA-IR, homeostatic model assessment of insulin resistance.

To investigate whether the depletion of Cav-1 in β cells could improve blood glucose levels in mice under HFD, pre-exposure KO of β-Cav-1 mice (KO group) was generated as described in our previous study (33). Obviously, that knocking out of Cav-1 did not induce significant differences in basal blood glucose (WT + CD/3.93 ± 0.39 *versus* KO + CD/4.38 ± 1.03 mmol/l; *p* = 0.44) (Fig. 2*A*) and insulin levels (WT + CD/0.32 ± 0.09 *versus* KO + CD/0.29 ± 0.01 ng/ml; *p* = 0.56) (Fig. 2*B*) in mice on CD. As expected, basal glucose (WT + CD/3.93 ± 0.39 *versus* WT + HFD/10.50 ± 1.27 mmol/l; *p* < 0.001) and insulin levels (WT + CD/0.32 ± 0.09 *versus* WT + HFD/1.60 ± 0.28 ng/ml; *p* < 0.001) weresignificantly increased in WT mice subjected to HFD 16 weeks later. Interestingly, the basal glucose levels (WT + HFD/10.50 ± 1.27 *versus* KO + HFD/6.80 ± 0.78 mmol/l; *p* = 0.003) and insulin levels (WT + HFD/1.60 ± 0.28 *versus* KO + HFD/0.31 ± 0.07 ng/ml; *p* < 0.001) in KO + HFD mice were notably lower compared to those in WT + HFD mice.

Similarly, the adjusted HOMA-IR index increased significantly in WT + HFD mice compared to WT + CD mice (WT + CD/1.18 ± 0.35 *versus* WT + HFD/15.94 ± 3.67; *p* < 0.001) (Fig. 2*C*). For mice on CD, no notable difference was observed in adjusted HOMA-IR between WT mice and KO mice (WT + CD/1.18 ± 0.35 *versus* KO + CD/1.19 ± 0.26; *p* = 0.95). While for mice on HFD, there was a significant decrease in HOMA-IR in KO + HFD mice compared to WT + HFD mice (WT + HFD/15.94 ± 3.67 *versus* KO + HFD/1.93 ± 0.26; *p* < 0.001). Furthermore, the augment of C-peptide (CP) levels on HFD (WT + CD/5.90 ± 0.53 *versus* WT + HFD/7.29 ± 0.25 ng/ml; *p* < 0.01) was reversed after Cav-1 depletion (WT + HFD/7.29 ± 0.25 *versus* KO + HFD/5.35 ± 0.45 ng/ml; *p* < 0.001) (Fig. 2*D*). Consistently, there was no significant difference between KO + CD and KO + HFD groups of mice, suggesting normal insulin secretion of islets β cells after Cav-1 depletion. Taken together, these results suggested that β-Cav-1 KO significantly prevented blood glucose increase and insulin resistance in HFD mice.

### Pre-exposure KO of β-Cav-1 improves glucose tolerance in mice under HFD

**Figure 3:**
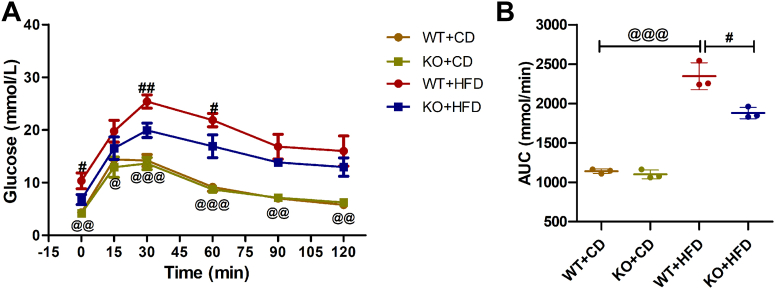
**IPGTT in pre-exposure knockout of β-Cav-1 and WT mice.** The response kinetics (*A*) and the AUC (*B*) for each group were calculated and plotted in pre-exposure knockout studies. Data presented are averages (*A*) and individual data points (*B*) from results obtained for each group separately. Data here are representative of three independent experiments and represent the mean (n = 3) ± SD. ^@^ = *p* < 0.05, ^@@^ = *p* < 0.01 and ^@@@^ = *p* < 0.001 between WT + CD group and WT + HFD group; ^#^ = *p* < 0.05 and ^##^ = *p* < 0.01 between WT + HFD group and KO + HFD group. IPGTT, intraperitoneal glucose tolerance test; AUC, area under the curve; CD, control diet; HFD, high-fat diet.

Intraperitoneal glucose tolerance test (IPGTT) assays were then carried out to evaluate the impact of Cav-1 depletion on stabilizing glucose homeostasis under a glucose load. Before exposure to HFD, there were no significant differences in glucose levels between WT and KO mice after glucose injection (Fig. 3*A*). In contrast, HFD significantly increased blood glucose levels in both WT and KO mice. However, glucose levels in β-Cav-1 KO mice on HFD were significantly lower than in WT mice on HFD at 0 min (baseline) (KO + HFD/6.80 ± 0.95 *versus* WT + HFD/10.33 ± 1.50 mmol/l; *p* = 0.026), at 30 min (KO + HFD/19.93 ± 1.36 *versus* WT + HFD/25.40 ± 1.28 mmol/l; *p* = 0.007) and at 60 min (KO + HFD/16.9 ± 2.16 *versus* WT + HFD/21.87 ± 1.27 mmol/l; *p* = 0.026) after glucose injection. In addition, the area under the curve (AUC) for WT + HFD mice was significantly higher compared to WT + CD mice (WT + HFD/2346 ± 170.46 *versus* WT + CD/1139.67 ± 22.74 mmol/min; *p* < 0.001 (Fig. 3*B*). Similarly, the increased AUC caused by HFD was at least partly reversed in β-Cav-1 KO + HFD mice compared to WT + HFD mice (KO + HFD/1879 ± 71.12 *versus* WT + HFD/2346 ± 170.46 mmol/min; *p* = 0.012). These results clearly suggested that Cav-1 KO improved glucose tolerance impaired by HFD.

### Post-exposure KO of β-Cav-1 similarly decreased blood glucose levels, HOMA-IR, and improved glucose tolerance in HFD-fed mice

**Figure 4:**
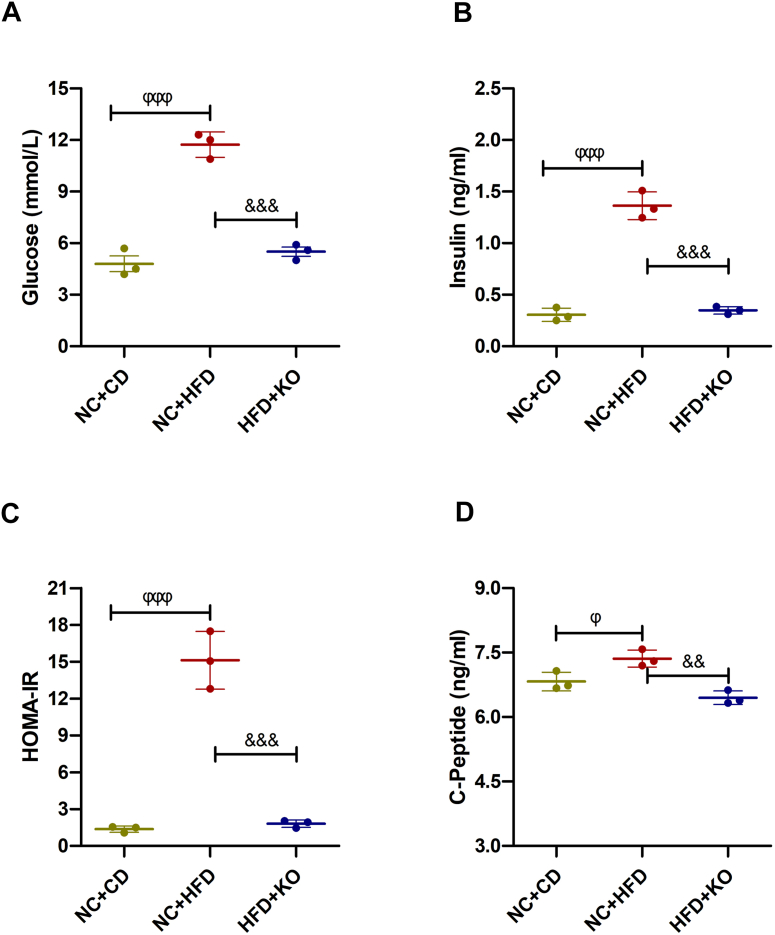
**Measurement of serum glucose, insulin, HOMA-IR, and C-peptide in post-exposure Cav-1 knockout study.** Basal glucose level (*A*), basal insulin secretion (*B*), HOMA-IR (*C*), and C-peptide levels (*D*) in negative control (NC) and post-exposure knockout of Cav-1 mice fed a control diet (CD) or high-fat diet (HFD) were measured according to each kit’s instructions. Data are representative of three independent experiments and represent the mean (n = 3) ± SD. ^ϕϕϕ^ = *p* < 0.001 between NC + =CD group and NC + HFD group; ^&&&^ = *p* < 0.001 between NC + HFD group and HFD + KO group. HOMA-IR, homeostatic model assessment of insulin resistance; Cav-1, caveolin-1.

To investigate whether KO of β-Cav-1 after HFD feeding could similarly ameliorate blood glucose levels, post-exposure KO of β-Cav-1 mice (HFD + KO group) was generated as described in our previous study (33). Consistent with the front results, negative control (NC) + HFD group of mice exhibited higher levels of basal glucose (*p* < 0.001), insulin (*p* < 0.001), and HOMA-IR values (*p* < 0.001), compared to the NC + CD group (Fig. 4, *A*–*C*). Interestingly, KO of β-cell Cav-1 in HFD-fed mice partially reversed the elevation of glucose levels (HFD + KO/5.50 ± 0.46 *versus* NC + HFD/11.73 ± 0.74 mmol/l; *p* < 0.001), insulin levels (HFD + KO/0.35 ± 0.04 mmol/l *versus* NC + HFD/1.36 ± 0.13 mmol/l; *p* < 0.001), and HOMA-IR (HFD + KO/1.81 ± 0.30 mmol/l *versus* NC + HFD/15.12 ± 2.35 mmol/l; *p* < 0.001). Again, CP levels also increased significantly under HFD compared CD in NC mice (NC + CD/6.83 ± 0.22 *versus* NC + HFD/7.36 ± 0.20 ng/ml; *p* < 0.05), but reversed after the KO of β-Cav-1 in HFD + KO mice compared to NC + HFD mice (NC + HFD/7.36 ± 0.20 *versus* HFD + KO/6.45 ± 0.16 ng/ml; *p* < 0.01) (Fig. 4*D*). These findings suggest that post-exposure KO of β-Cav-1 in HFD-fed mice also decreases blood glucose levels and improves insulin resistance.

**Figure 5:**
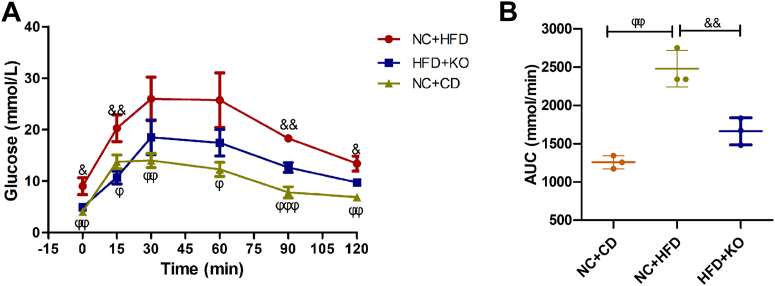
**IPGTT in post-exposure knockout of β-Cav-1 and NC mice.** The response kinetics (*A*) and the AUC (*B*) for each group were calculated and plotted in post-exposure knockout studies. Data presented are averages for *panel* (*A*) and individual data points for *panel* (*B*) obtained separately from each group. Data here are representative of three independent experiments and represent the mean (n = 3) ± SD. ^Φ^ = *p* < 0.05 and ^ϕϕ^ = *p* < 0.01 between NC + CD group and NC + HFD group; ^&^ = *p* < 0.05, ^&&^ = *p* < 0.01 and ^&&&^ = *p* < 0.001 between NC + HFD group and HFD + KO group. HOMA-IR, homeostatic model assessment of insulin resistance. AUC, area under the curve. IPGTT, intraperitoneal glucose tolerance test; AUC, area under the curve; CD, control diet; HFD, high-fat diet.

Furthermore, IPGTT was carried out to assess the effect of post-exposure KO of β-Cav-1 on glucose tolerance regulation. As expected, HFD caused an increase in blood glucose levels similar to pre-exposure experiment (Fig. 4*A*). Notably, compared to the NC + HFD group mice, the post-exposure KO of β-Cav-1 in the HFD + KO group mice significantly reduced the elevated blood glucose levels at various time points: at 0 min (NC + HFD/9.00 ± 1.65 *versus* HFD + KO/4.90 ± 0.17 mmol/l; *p* = 0.013), at 15 min (NC + HFD/20.27 ± 2.61 *versus* HFD + KO/10.70 ± 1.25 mmol/l; *p* = 0.005), at 90 min (NC + HFD/18.30 ± 0.76 *versus* HFD + KO/12.63 ± 0.95 mmol/l; *p* = 0.001), and at 120 min (NC + HFD/13.40 ± 1.45 *versus* HFD + KO/9.73 ± 0.60 mmol/l; *p* = 0.016) after glucose injection. Furthermore, the AUC in NC + HFD mice was significantly higher compared to NC + CD mice (NC + HFD/2478 ± 237.30 *versus* NC + CD/1256 ± 86.01 mmol/min; *p* = 0.001) (Fig. 5*B*). Again, the increased AUC caused by HFD was significantly reduced in HFD + KO mice compared to NC + HFD mice (HFD + KO/1662 ± 176.73 *versus* NC + HFD/2478 ± 237.30 mmol/min; *p* = 0.009).

Together, these results suggest that post-exposure KO of β-Cav-1 in HFD-fed mice exhibited a similar improvement in glucose tolerance as observed in pre-exposure KO of β-Cav-1.

### Cav-1 depletion protected PA-induced β-cell apoptosis through p38 MAPK) pathways

**Figure 6:**
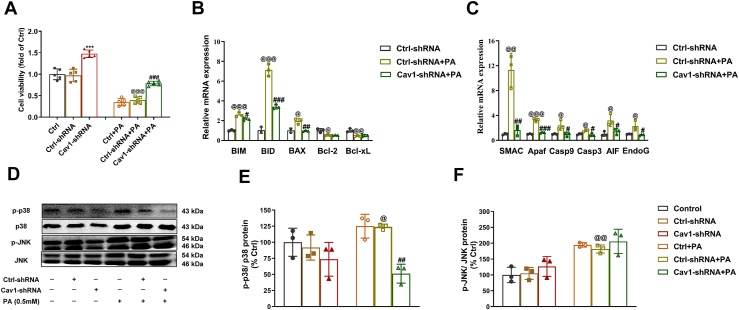
**Caveolin-1 depletion increased viability of β cells through modulation of apoptosis related genes and p38 MAPK pathway.***A*, MTT assay in NIT-1 pancreatic β cells. *B* and *C*, RT-PCR analysis of the expression of mitochondria-dependent molecule genes. *D*, Western blot analysis of p38 phosphorylation, total p38, JNK phosphorylation, and total JNK. *E* and *F*, quantification analysis of the ratios of phosphorylated p38 to total p38, and the ratios of phosphorylated JNK to total JNK (including p46 and p54 JNK isoforms) based on Western blot results. Data are representative of at least three independent experiments (n = 5, mean ± SD). ∗∗∗ = *p* < 0.001 between Cav1-shRNA groups and Ctrl-shRNA groups; ^#^ = *p* < 0.05, ^##^ = *p* < 0.01 and ^###^ = *p* < 0.001 between Cav1-shRNA + PA group and Ctrl-shRNA + PA group; ^@^ = *p* < 0.05, ^@@^ = *p* < 0.01 and ^@@@^ = *p* < 0.001 between Ctrl-shRNA + PA groups and Ctrl-shRNA groups. MAPK, mitogen-activated protein kinase; 3-(4,5-dimethylthiazole-2-yl)-2,5-diphenyl tetrazolium bromide; JNK, c-jun NH2-terminal kinase; Cav-1, Caveolin-1; PA, palmitate.

We previously reported that the depletion of Cav-1 inhibited PA-induced cell apoptosis in the β cell line NIT-1 (24). Here, we also found that Cav-1 depletion increased cell viability of NIT-1 cells (Fig. 6*A*). To further explore the cytoprotective mechanisms, we performed RT-PCR analysis on mitochondria-dependent molecules involved in the apoptotic process. Following PA administration, there was a clear upregulation of mRNAs of proapoptotic proteins BIM, BID, and BCL-2-associated X protein (BAX), along with downregulation of mRNAs of antiapoptotic proteins BCL-2 and Bcl-xL (Fig. 6*B*). Meanwhile, in response to apoptotic stimulation (PA treatment), mRNA expression of caspase-dependent cell death factors, including the second mitochondria-derived activator of caspase (SMAC), apoptotic protease activating factor (Apaf), Caspase-9 (Casp9), and Caspase-3 (Casp3), along with caspase-independent cell-death-inducing factors, including apoptosis-inducing factor (AIF), Apaf, and EndoG were all elevated (Fig. 6*C*). However, after Cav-1 depletion, the gene expression levels of proapopototic molecules BIM, BID, SMAC, Apaf, Caspase9, Caspase3, AIF, and EndoG were all significantly decreased, while antiapoptotic genes Bcl-2 and Bcl-xL remained unchanged (Fig. 6, *B* and *C*). These data suggested that Cav-1 depletion rescued high lipid stressing cells through improvement of apoptosis.

To further investigate the pathways involved in the protective effect of Cav-1 depletion on β-cell viability, we carried out Western blot to measure the expression of MAPK proteins p38 and c-jun NH2-terminal kinase (JNK (Fig. 6*D*). We found that exposure of NIT-1 cells to palmitic acid (PA) resulted in a 1.25-fold and 2-fold increase in the phosphorylation of p38 and JNK, respectively, compared to the vector control groups (*p* < 0.05 and *p* < 0.01, respectively) (Fig. 6, *E* and *F*). However, silencing Cav-1 significantly reversed the PA-induced increase of p38 phosphorylation (p-p38) (*p* < 0.01) (Fig. 6*E*), but not JNK phosphorylation (Fig. 6*F*). These results suggested that Cav-1 depletion protected β cells from PA-induced cell death primarily through the p38 MAPK pathway, but not JNK pathway.

### Cav-1 depletion ameliorated PA-induced autophagy dysregulation

**Figure 7:**
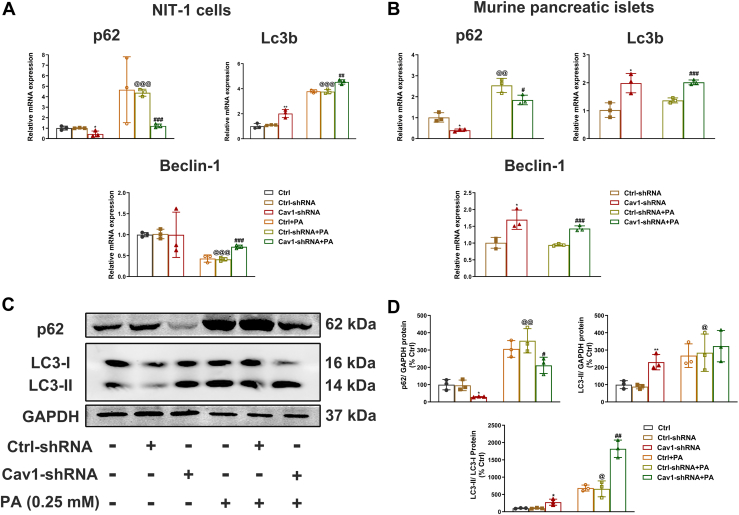
**Expressions of autophagy-related genes in NIT-1 cells and mice pancreatic β cells with or without Cav-1 depletion.** mRNA expression levels of autophagy-related genes Lc3b, p62, and Beclin-1 were measured by qPCR in NIT-1 cells (*A*) and islets (*B*). Protein levels for LC3-I, LC3-II, and p62 were determined by Western blot (*C*) and standardized to expression of GAPDH with or without PA treatment in NIT-1 β cells (*D*). Data here were representatives of three independent experiments (n = 3, mean ± SD). ∗ = *p* < 0.05, and ∗∗ = *p* < 0.01 between Cav-1-shRNA group and Ctrl-shRNA group; ^#^ = *p* < 0.05, ^##^ = *p* < 0.01 and ^###^ = *p* < 0.001 between Cav-1-shRNA + PA group and Ctrl-shRNA + PA group; ^@^ = *p* < 0.05, ^@@^ = *p* < 0.01 and ^@@@^ = *p* < 0.001 between Ctrl-shRNA + PA group and Ctrl-shRNA group. Cav-1, Caveolin-1; PA, palmitate.

Considering that autophagy effectively protects pancreatic β-cells against PA-induced dysfunction and apoptosis, we then investigated the expression of autophagy-specific genes using RT-PCR and Western blot assays. We found that the mRNA levels of the autophagy marker Lc3b (Fig. 7, *A* and *B*) and the protein level of the autophagosome expansion and maturation regulator LC3-II (Fig. 7*C*), along with the ratio of LC3-II to LC3-I protein expression (Fig. 7*D*), were significantly increased following PA treatment in both NIT-1 cells and mouse islets. Notably, Cav-1 depletion further elevated Lc3b mRNA levels and the ratios of LC3-II to LC3-I protein expression compared to control NIT-1 cells or mouse islets after PA stimulation. Besides, RT-PCR analysis also revealed that in cells treated with PA, the upstream gene Beclin-1, which is involved in autophagy induction, was downregulated compared to control cells (Fig. 7, *A* and *B*) (^@@@^*p* < 0.001). In addition, PA treatment enhanced the accumulation of the autophagic substrate p62 in NIT-1 cells and islets (Fig. 7, *A*–*D*). However, these lipotoxic effects were all reversed following Cav-1 depletion, thereby promoting the proper progression of autophagy. Taken together, these findings indicated that Cav-1 depletion mitigated PA-induced autophagy dysregulation, which ultimately contributed to the protection of pancreatic β-cell function.

### Cav-1 depletion negatively regulates the mTOR pathway in NIT-1 pancreatic β cells

**Figure 8:**
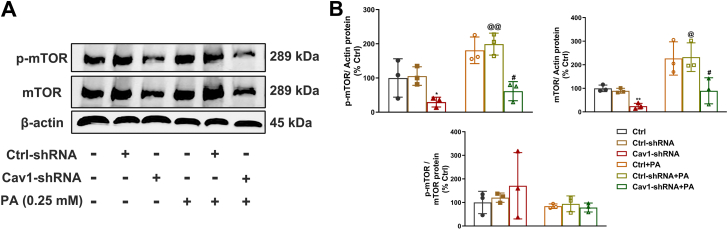
**Cav-1 depletion promoted autophagy *via* regulation of the mTOR pathway in pancreatic 46 cells.** Protein expression of phosphorylation (Ser2448) of mTOR and total mTOR were determined by Western blot analysis (*A*) and standardized to the expression of β-Actin, with or without PA treatment in NIT-1 β cells (*B*). Data here were representatives of three independent experiments (n = 3, mean ± SD). ∗ = *p* < 0.05 and ∗∗ = *p* < 0.01 between Cav-1-shRNA group and Ctrl-shRNA group; ^#^ = *p* < 0.05 between Cav-1-shRNA + PA group and Ctrl-shRNA + PA group; ^@^ = *p* < 0.05 and ^@@^ = *p* < 0.01 between Ctrl-shRNA + PA group and Ctrl-shRNA group. Cav-1, Caveolin-1; PA, palmitate.

To further elucidate the underlying mechanisms driving Cav-1 depletion-mediated autophagy regulation, we measured mTOR protein expression by Western blot (Fig. 8*A*). PA treatment promoted the expression of phosphorylated mTOR and total mTOR (Fig. 8*B*). These results revealed that elevated levels of phosphorylated mTOR and total mTOR in PA treatment groups were drastically reversed by Cav-1 depletion, suggesting the restoration of autophagy function (Fig. 8*B*).

## Discussion

Our previous *in vitro* study revealed that Cav-1 depletion protected β cells against PA-induced apoptosis (24). Further research demonstrated that deficiency of Cav-1 alleviated intracellular lipid accumulation and inflammation in pancreatic β cells (33). Therefore, further investigation into the phenotype associated with glucose homeostasis regulation *in vivo* and the underlying mechanisms by which Cav-1 influences β-cell function is in urgent. In this study, we discovered that KO of β-cell Cav-1 effectively prevented the elevation of blood glucose and HOMA-IR, improved insulin resistance in HFD mice. Furthermore, our results indicated that Cav-1 depletion protected β cells from PA-induced apoptosis through p38 MAPK pathways, and promoted β-cell autophagy through mTOR signaling pathway.

In present study, we observed that depletion of Cav-1 could augment islets size independent of diet type and this augmentation could be further strengthened under lipid stressing. Importantly, the islets circularity of mice suffering from HFD or/and Cav-1 KO was barely changed (Fig. 1). These results are partially similar with Paloma Lillo Urzúa’s findings that islet size increased significantly with HFD for systematic CAV1 KO mice (21). However, Paloma’s study presented obviously few circular islets in CAV1 KO mice compared to WT mice, suggesting morphological abnormalities. In contrast, we did not observe significant change on islet shape. Since type 2 diabetes mellitus patient presented smaller and less spherical shaped islets (34), our results, appearing larger and more spherical, suggested that our β cell-specific (rather than systematic) KO of Cav-1 contributed to much healthier islets. It's not surprising that the enlarged islets might contain more β cells to maintain blood glucose balance under HFD pressure. This result was consistent with our previous *in vitro* study that the depletion of Cav-1 predominantly inhibited cell cycle inhibitors and enhanced the expression of cyclins, resulting in increased proliferation of β cells (24).

For the glucose management, we observed that the HFD increased mice blood glucose (Figs. 2*A* and 4*A*) similar to Paloma’s study (21). In addition, we also found HFD induced significant increase in the insulin levels, as well as adjusted HOMA-IR values and blood levels of CP (Figs. 2, *B*–*D* and 4, *B*–*D*). The reason for increased insulin and CP levels in mice on HFD might due to a compensatory response to impaired insulin utilization in a lipotoxic environment. Intriguingly, we found that pre-exposure and post-exposure KO of β-Cav-1 reversed lipotoxicity-induced elevation of glucose, insulin, and HOMA-IR (Figs. 2, *A*–*C* and 4, *A*–*C*). Furthermore, β-cell Cav-1 KO mice exhibited normal CP levels similar to control mice (Figs. 2*D* and 4*D*). Given that elevated levels of CP and insulin in the bloodstream were indicatives of insulin resistance (35, 36), our results suggested that β-cell specific Cav-1 KO protected mice from insulin resistance.

Previous studies reported that systematic depletion of Cav-1 lead to serious insulin resistance and poor metabolic status, probably resulting from obstruction of insulin utility and abnormalities of intracellular insulin signaling pathways in various tissues (17, 18, 21, 22). In our study, mice with β-Cav-1 KO did not cause metabolic abnormalities in tissues other than pancreatic islets, which could better reflect the specific role of Cav-1 *in vivo* concerning insulin and glucose regulation under prodiabetogenic conditions. In the same way, diminished glucose tolerance observed in mice on HFD was significantly improved in both pre-exposure and post-exposure mice (Figs. 3 and 5).

To further investigate the mechanisms underlying Cav-1 depletion improved β-cell dysfunction under HFD, we constructed β-cell specific Cav-1 KO models of NIT-1 cells and isolated islets as described in our previous studies (24, 33). As expected, we observed increased survival rates in Cav-1 depleted β cells under PA treatment (Fig. 6*A*), consistent with what we found in the earlier study (24). Further mechanism study revealed that Cav-1 depletion exerted anti-apoptotic functions through the modulation of gene expressions of several mitochondria-related apoptotic regulators. Particularly, the protection of β-cell apoptosis was clearly through the suppression of gene expressions of pro-apoptotic proteins (Bim, Bid, and Bax). The reason might be associated with suppression of BAX expression through Cav-1 depletion, which inhibited the initial step in the induction of apoptosis (37, 38). It is known that Bcl-2 and Bcl-xL are negatively regulated by BIM and BID (37, 38). Since Bim and Bid expression did not increase in PA-treated Cav-1-depleted β-cells, it might be inferred that the existing Bcl-2 and Bcl-xL (albeit at low concentrations) would function effectively enough to achieve apoptosis protection. Furthermore, Cav-1 depletion blocked the second step in apoptotic cascade (mitochondrial membrane leakage) accompanied by down-regulation of SMAC, Apaf, Caspase nine, and Caspases 3. Meanwhile, gene expressions of caspase-independent cell-death-inducing factors (AIF and endonuclease G (EndoG)), which were capable to promote chromatin condensation and DNA fragmentation during the execution phase of apoptosis, were also decreased in β cells with Cav-1 depletion under PA treatment. Since mitochondria plays a central role in β-cell apoptosis and glucose-stimulated insulin secretion, the observed partial protection against PA-induced apoptosis and dysfunction could be explained by downregulated expression of mitochondria-associated apoptotic molecules in Cav-1 silenced NIT-1 cells. Interestingly, it appears that the protective effect of Cav-1 depletion on PA-treated NIT-1 cells is primarily anti-apoptotic rather than pro-survival or pro-proliferative. Future studies are encouraged to explore mechanisms underlying Cav-1 depletion resulted in suppression of mitochondria-related apoptosis.

To date, the relationship between CAV1 and MAPKs has been reported in various cell types (21, 24, 39, 40). In MAPK pathways, JNK and p38 activation induced by FFAs in β cells is known to be pro-apoptotic, while ERK1/2 activation is considered anti-apoptotic (41). In this study, we observed significant activation of JNK and p38 in NIT-1 cells following PA treatment, consistent with previous findings (21). Notably, knockdown of CAV1 diminished the phosphorylation of p38, but not that of JNK under PA treatment (Fig. 6). However, further studies using loss- and/or gain-of-function strategies for p38 protein are necessary to validate the involvement and exclusiveness of p38 pathway in Cav-1 mediated β cell protection.

Autophagy is an intracellular lysosomal degradation pathway that is activated in cells under various stresses, playing a pivotal role in determining the fate of islet β cells (42, 43, 44). Recent studies have revealed that Cav-1 participated in the regulation of autophagy in various diseases (25, 45). To date, various mechanisms underlying Cav-1-mediated autophagy regulation were proposed. Jihoon Nah demonstrated that phosphorylated Cav-1 localized beclin-1 to the mitochondria, activating autophagy under oxidative stress in HeLa cells (46). Others demonstrated that Cav-1 induced oncogenic autophagy in renal cells by regulating the LC3-II protein, a marker of early-phase autophagy (31, 47). In this situation, p62, a substrate of autophagy that binds to LC3-II, was involved in targeting cargo to autophagosomes for degradation in the early phase and then be cleared during the late phases of autophagy (48, 49). In contrast, p62 accumulation indicated an impaired autophagy process (48).

In present study, we observed impaired autophagy in response to PA treatment, as evidenced by p62 accumulation and beclin-1 (involved in autophagy induction) reduction in β cells (Fig. 7). In contrast, KO of Cav-1 in mice with PA treatment resulted in increased formation of autophagolysosomes, characterized with the decrease of p62 to elevated Lc3b, as well as partially restoration of Beclin-1 expression (Fig. 7). Collectively, our results suggested that Cav-1 depletion alleviated PA-induced impairment of autophagy. It is well researched that mTOR is a major negative regulator of autophagy (27, 50, 51). Cav-1 has been reported to interact with and stabilize mTOR complex components. Consistent with this notion, Cav-1 deficiency induces cardiac dysfunction by suppressing autophagy *via* the AdipoR1-AMPK-mTOR pathway (52). Similarly, basic fibroblast growth factor (bFGF) deficiency has been shown to suppress Cav-1, thereby inhibiting mTOR signaling and exacerbating cognitive deficits after acute ischemic stroke in juvenile rats. Notably, knockdown of Cav1 in the hippocampus likewise attenuates mTOR signaling (53). In this study, we found that PA treatment obviously increased the expression of mTOR and its activation status as shown by phosphorylation of mTOR. However, Cav-1 depletion significantly decreased the expressions of both mTOR and phosphorylation of mTOR to the levels without PA stressing (Fig. 8). These findings are consistent with earlier studies showing that Cav-1 deficiency promotes autophagy through the Akt/mTOR signaling pathway (27). Notably, Cav-1 depletion did not significantly alter the p-mTOR/mTOR ratio with or without PA treatment, implying Cav-1 likely regulated other aspects of mTOR, but not its phosphorylation (Fig. 8). Mechanistically, Cav-1 functions as a scaffolding protein within caveolae/lipid rafts, where it recruits and anchors signaling molecules. Consequently, silencing of Cav-1 may disrupt the structural integrity of these microdomains, leading to enhanced mTOR protein degradation or reduced protein stability. Our findings suggested that Cav-1 depletion improved β-cell autophagy through the mTOR signaling pathway. In the future, more studies are encouraged to study the influence of Cav-1 depletion to β-cell dysfunction. In particular, mechanism studies are required to elucidate how Cav-1 depletion inhibits the mTOR pathway in detail. Finally, *in vivo* studies using animal models are also needed to confirm the protective effects of Cav-1 inhibition in lipid stressing.

In summary, our study found that β-Cav-1 KO mice exhibited an altered glucose metabolism, with reduced insulin resistance and improved glucose tolerance, as well as improved cell viability. Mechanism studies revealed that Cav-1 depletion protected β cells from PA-induced apoptosis primarily through p38 MAPK signaling pathway. Further investigation also suggested that improved apoptosis was attributed to enhanced autophagy through the involvement of mTOR signaling pathway. Our results provide valuable insights into the potential utilization of Cav-1 inhibition to improve β-cell function in effective diabetes therapies in the future.

## Experimental procedures

### Animals

All animal procedures were approved by the Institute Animal Care and Use Committee (IACUC) at the Animal Ethics Committees of Sun Yat-Sen University (Approval number: SYSU-IACUC-2020 to 000216). We generated Cre + flox/flox mice by crossing the conditional KO flox/flox mice (Cyagen Biosciences) with Cre mice (Genotype: B6. Cg-Tg (Ins1-cre/ERT) 1Lphi/J; Jackson Laboratory). Then, Cre + flox/flox mice were given tamoxifen (i.p.) (MedChemExpress Company) to induce β-Cav-1 KO mice, as our previous study described (33). Male flox/flox mice aged 8 weeks that received tamoxifen were used as the WT group, while Cre + flox/flox mice that did not receive tamoxifen served as the NC. Subsequently, mice in all groups (including NC group, WT group mice, and KO group) were fed by either CD (NC + CD group, WT + CD group, KO + CD group) or HFD (NC + HFD group, WT + HFD group, KO + HFD group). Here, mice with the β-Cav-1 KO occurred prior to HFD (KO + HFD mice) were used for pre-exposure KO studies, while KO occurred after HFD (HFD + KO mice) were used for the post-exposure KO studies. The flow chart of the *in vivo* experimental design is presented in our previous study (33).

All mice were housed under a standard 12:12-h light-dark cycle at a constant temperature of 21 °C, with free access to water and a commercial CD (4% fat (wt/wt), Guangdong Medical Laboratory Animal Center) or water and HFD (60% fat, D12492, Research Diets) throughout the duration of the experiment.

### IPGTT

At the end of each *in vivo* experiment, all mice were subjected to an 8 h of fasting and then assessed for IPGTT. Firstly, all mice were weighed, and infused intraperitoneally (i.p.) at a dose of 2*g* glucose per kg mouse body weight. Next, caudal capillary blood samples were collected consecutively at 0 min, 15 min, 30 min, 60 min, 90 min, and 120 min post-infusion. Finally, blood glucose levels were quantified using the Accutrend Glucose Test Strips system (Roche Diagnostics, Indianapolis, IN, USA). The AUCs for the IPGTT were calculated using GraphPad Prism 5 software (GraphPad Software).

### Blood samples

All mice underwent an 8-h fasting period, after which a sample of caudal capillary blood was collected to measure fasting blood glucose levels (basal). Then, all mice were anesthetized by ether, and sacrificed for blood sample collection. All procedures were carried out in accordance with the National Institutes of Health guidelines. Plasma was obtained by centrifuging the samples at 2000*g* for 10 min in a refrigerated centrifuge. Finally, insulin levels (Mouse Ultrasensitive Insulin ELISA, ALPCO) and CP levels (Mouse CP ELISA Kit, ELK Biotechnology Co., Ltd), Bio Vision) were measured (basal). The HOMA-IR adjusted for the mice was calculated using the following formula: basal glucose (mmol/l) × basal insulin (ng/ml) × 21.2/22.51.

### Histological staining and study of islet morphology

After each *in vivo* experiment, the pancreas was extracted from male mice, then fixed in a 10% p-formaldehyde in a neutral buffer solution overnight and embedded in paraffin. Hematoxylin and Eosin solutions were used to assess morphological changes in the pancreatic islets. The size and circularity of the islets were analyzed using ImageJ software (the National Institutes of Health). Here, the circularity of the islets was calculated using the following equation: circularity = 4 π (area/perimeter^2^), and a value of 1.0 indicates a perfect circle (54).

### Mouse pancreatic islets isolation

Male C57BL/6J mice of aged 8 to 10 weeks were procured from the Guangzhou Medical Laboratory Animal Center. The mice were housed under a 12 h light/dark cycle with 50% humidity at 23 °C and fed a normal diet (4% fat [wt/wt]) before islets isolation. All animal-based experimental procedures performed in this study were reviewed and approved by The Institutional Animal Care and Use Committees (IACUC) of Sun Yat-Sen University (Approval number: SYSU-IACUC-2020 to 000216) and conducted in accordance with local and international legislation regarding the well-being of laboratory animals. Procedures for mouse pancreatic islets isolation and dispersion have been described with detail in our previously study (24). Briefly, Mice were anesthetized with chloral hydrate and then sacrificed by cervical dislocation. After ampulla clamped, collagenase P (0.5 mg/ml, Roche Diagnostics) in Hanks' balanced salt solution (HBSS, Life Technologies) was injected into mouse pancreas through the common bile duct. Next, the pancreas was quickly removed for digestion at 37 °C in a water bath. Five minutes later, digestion was terminated with 3 ml cold HBSS containing 10% fetal bovine serum (FBS). Then, islets were purified by centrifugation at 500*g* for 0.5 min at 4 °C and rinsed three times with cold HBSS containing 10% FBS. Finally, the intact islets were handpicked under a stereomicroscope from the digest solution.

### Cell culture and cell viability assay

Mouse NIT-1 pancreatic β cells were purchased from American Type Culture Collection (ATCC) and cultured in RPMI-1640 medium (Life Technologies) supplemented with 10% FBS (Life Technologies) and penicillin/streptomycin (100 mg/ml, Invitrogen) at 37 °C with 5% CO_2_. All cells were used between passages of 10 and 38. The RPMI-1640 medium containing 0.25 mM PA (Sigma-Aldrich) and 1% bovine serum albumin (EMD Millipore Corp) vehicle control for experiments was prepared as described previously (24, 33).

For Cav-1 depletion, short hairpin sequences against either Cav-1 gene or the scrambled shRNA were cloned into the EGFP-labeled lentiviral vector GV248 (GENECHEM) and transfected as described previously (24, 33). Briefly, NIT-1 cells were seeded in plate well and grown to a density of 70 to 80%. Next, NIT-1 cells were transfected at a multiplicity of infection of 20 with Cav-1 shRNA (Cav1-RNAi37949–1: ACGTGGTCAAGATTGACTT) or non-targeting shRNA (Ctrl-shRNA: TTCTCCGAACGTGTCACGT) and cultured for 12 h. For mouse islets, transfection was carried out immediately after isolation at a multiplicity of infection of 100 and cultured for 24 h. Then, transfection mixtures was replaced with regular medium and incubated at 37 °C for 72 h. Finally, Cav-1 protein levels in NIT-1 cells were measured by Western blotting, and islet cells were measured by immunofluorescence assay to confirm effective gene knockdown, as our previous study described (24).

The viability of treated NIT-1 cells was assessed by 3-(4,5-dimethylthiazole-2-yl)-2,5-diphenyl tetrazolium bromide assay (Roche Diagnostics) according to manufacturer’s instructions. The absorbance values of the samples were measured at 450 nm by a microplate reader (BioTek).

### Quantitative real-time PCR

Total RNA from cells was extracted with TRIzol reagent (Sigma-Aldrich) according to the manufacturer’s instructions. cDNA was transcribed using PrimeScript RT reagent Kit (Cat. # RR047A; Takara Bio). mRNA levels were quantified using quantitative real-time PCR with the SYBR Premix Ex Taq II kit (Cat. #RR820A; Takara Bio Inc). mRNA level of β-actin was used as an endogenous control. The data were analyzed relative to controls. All assays were performed on the LightCycler 480II real-time PCR system (Roche Diagnostics). The details of primer sequences for qPCR are provided in Table S1.

### Western blot analysis

NIT-1 cells were homogenized in radioimmunoprecipitation assay buffer (Thermo Fisher Scientific). Nuclear extraction was performed using the EpiQuik Extraction Kit I (Epigentek Inc.) as previously described (24, 33). Protein concentration was quantified using a BCA protein estimation kit (Beyotime). Western Blot analysis of cell lysates was performed as described in earlier studies (24). Primary antibodies including these against Cav-1 (Cat. #3267), phosphorylated (p)-p38 MAPK(Thr180/Tyr182) (Cat. #9211), p38 MAPK (Cat. #9212), JNK phosphorylation (Cat. #4668), JNK (Cat. #9252), p62 Antibody (Cat. #5114), LC3I/II (Cat. #12741), Phospho-mTOR (Ser2448) (Cat. #5536), mTOR (Cat. #2972), β-actin (Cat. #8457), and GAPDH (Cat. #5174), all purchased from Cell Signaling Technology and diluted at 1: 1000. Parallel blotting of β-actin or GAPDH served as the internal control. Following the blotting procedure, membranes were incubated with secondary antibodies (Thermo Fisher Scientific; DyLight 800, 1:10000) for 1 h at room temperature. Signals were scanned using the Odyssey infrared imaging system (LI-COR Biosciences). Developed blots were then measured *via* absorbance using Image J (NIH) and normalized according to the expression of internal controls: GAPDH or β-actin.

### Statistical analysis

Quantitative data are presented as the Mean ± SD from three or more independent experiments. *In vivo* results, except for the IPGTT, are expressed as individual data points with their respective means. Statistical analyses were conducted using a two-tailed, unpaired Student’s *t* test, and one-way ANOVA followed by Tukey’s *post hoc* test using Graph Pad Prism 5 software (Graph Pad Software). A value of *p* < 0.05 was considered significant in data analysis.

## Data availability

The data of this article are all available in the article, in its supporting information files, and will be shared upon request to the corresponding author.

## Supporting information

This article contains supporting information.

## Conflict of interest

The authors declare that they have no conflicts of interest with the contents of this article.
