# Supplementary material for: Caveolin-1 deficiency improved glucose metabolism via modulation of β-cell autophagy in high-fat diet-fed mice
Source: J Biol Chem. 2026 Apr 22;302(6):111480. doi: 10.1016/j.jbc.2026.111480 (PMC13241726; doi:10.1016/j.jbc.2026.111480)
Supplement: Table S1 [file mmc1.docx]

Supplementary Table 1: Sequence information of quantitative PCR analysis

| GenBank Accession No. | Gene | Sequence (5’-3’) |
| --- | --- | --- |
| NM_007616.4 | Caveolin-1 | Forward: GGGACATCTCTACACTGTTCCCATC |
|  |  | Reverse: CTTCTGGTTCTGCAATCACATCTTC |
| NM_009754 | BIM | Forward: CCCGGAGATACGGATTGCAC |
|  |  | Reverse: GCCTCGCGGTAATCATTTGC |
| NM_007544.3 | BID | Forward: GCCTTGTCGTTCTCCATGTCT |
|  |  | Reverse: AATCATCCACAACATTGCCAGA |
| NM_007527 | BAX | Forward: TGAAGACAGGGGCCTTTTTG |
|  |  | Reverse: AATTCGCCGGAGACACTCG |
| NM_177410.2 | Bcl-2 | Forward: TCGCAGAGATGTCCAGTCAG |
|  |  | Reverse: CCTGAAGAGTTCCTCCACCA |
| NM_009743.4 | Bcl-xL | Forward: TGCAATCCGACTCCCAATA |
|  |  | Reverse: CGGAGAGCGTTCAGTGATCT |
| NM_023232.3 | SMAC | Forward: GGATGTGATTCCTGGCAGTT |
|  |  | Reverse: GCGGTTCCTATTGCTCAGAA |
| NM_009684.2 | Apaf | Forward: GAGAAAACCCTGAGGCACAA |
|  |  | Reverse: TAATTAAAGCGGCTGCTCGT |
| NM_015733 | Casp9 | Forward: TCCTGGTACATCGAGACCTTG |
|  |  | Reverse: AAGTCCCTTTCGCAGAAACAG |
| NM_009810 | Casp3 | Forward: TCTGACTGGAAAGCCGAAACT |
|  |  | Reverse: AGGGACTGGATGAACCACGAC |
| NM_012019 | AIF | Forward: CAAACTGGCGGACTGGAAATAGA |
|  |  | Reverse: AGGGGCGCTGGGAGGAAT |
| NM_007931 | EndoG | Forward: AATGCCTGGAACAACCTTGAGA |
|  |  | Reverse: CACATAGCACTTCCCATCAGCC |
| NM_011018.3 | P62 | Forward: ATCTTCTGGGCAAGGAGGAGG |
|  |  | Reverse: GCCATTGTCAGCTCCTCATCAC |
| NM_026160.4 | LC3b | Forward: CGTCCGAGAAGACCTTCAAGC |
|  |  | Reverse: CTCATGTTCACGTGGTCAGGC |
| NM_019584.3 | Beclin-1 | Forward: TCAGTACCAGCGGGAGTATAGTG |
|  |  | Reverse: GCTGTGCCAGATGTGGAAGGT |
| NM_007393.2 | β-Actin | Forward: TGTTACCAACTGGGACGACATG |
|  |  | Reverse: CTGGATGGCTACGTACATGGCT |
